# Obesity History and Daily Patterns of Physical Activity at Age 60–64 Years: Findings From the MRC National Survey of Health and Development

**DOI:** 10.1093/gerona/glw331

**Published:** 2017-02-18

**Authors:** Rachel Cooper, Lei Huang, Rebecca Hardy, Adina Crainiceanu, Tamara Harris, Jennifer A Schrack, Ciprian Crainiceanu, Diana Kuh

**Affiliations:** 1 MRC Unit for Lifelong Health and Ageing at UCL, London.; 2 Department of Biostatistics, Johns Hopkins Bloomberg School of Public Health, Baltimore, Maryland.; 3 Computer Science Department, United States Naval Academy, Annapolis, Maryland.; 4 Laboratory of Epidemiology and Population Sciences, National Institute on Aging, National Institutes of Health, Bethesda, Maryland.; 5 Department of Epidemiology, Johns Hopkins Bloomberg School of Public Health, Baltimore, Maryland.

**Keywords:** BMI, Daily patterns of activity, Aging, Birth cohort, Life course

## Abstract

**Background:**

The aim of this study was to investigate associations of current body mass index (BMI) and obesity history with daily patterns of physical activity.

**Methods:**

At age 60–64, participants from a British birth cohort study wore accelerometers for 5 days. Accelerometry counts were log-transformed and mean log-counts were used to derive a summary variable indicating total daily log-activity counts. Among those with complete data (*n* = 1,388) the associations of current BMI and age of first obesity were examined with: (a) total daily log-activity counts and (b) total log-activity counts in four segments of the day.

**Results:**

Higher current BMI and younger age at obesity were strongly associated with lower levels of total daily activity at age 60–64 even after adjustment for sex, socioeconomic factors, and health status. The fully-adjusted mean difference in total daily log-activity counts was −581.7 (95% confidence interval: −757.2, −406.3) when comparing BMI ≥35 kg/m^2^ with <25 kg/m^2^, representing an 18.4% difference. Participants who had been obese since early adulthood had the lowest levels of activity (mean difference in total daily log-activity counts was −413.1 (−638.1, −188.2) when comparing those who were obese by age 26 or 36 with those who were never obese, representing a 13.1% difference).

**Conclusions:**

Obese older adults may require targeted interventions and additional support to improve their daily activity levels. As younger generations with greater lifetime exposure to obesity reach old age the proportion of adults achieving sufficient levels of activity to realize its associated health benefits is likely to decline.

Recent evidence suggests that it may never be too late in life to intervene to modify physical activity (PA) levels and observe beneficial effects on health and function (1). Increasing PA levels in older populations therefore has the potential to address the public health challenges that global population aging presents (2). Research to identify modifiable factors associated with PA at older ages is therefore essential and has recently been enhanced by the advent of accelerometers which provide a wider range of PA parameters (3–5), including daily patterns, and are now widely used in older populations (6).

Health-related factors typically considered as outcomes of PA could be important targets for intervention when identifying opportunities to improve levels of PA in older populations. Body mass index (BMI) is one factor that may influence daily patterns of PA. While some existing studies have examined the associations of BMI with monitored levels of PA, most have focused on younger populations or populations covering a wide age range (7–14). As the majority of these studies have been cross-sectional, the potential influence of weight history has yet to be considered.

With increases in the global prevalence of obesity there is growing public health concern regarding the increasing proportion of the population reaching old age that have lived more of their lives obese. It is expected that greater lifetime exposure to obesity will be associated with less favorable patterns of PA but the associations of weight history with daily quantities, patterns, and trends of PA have not yet been investigated. To this end, this article aims to investigate the associations of current BMI and obesity history with daily patterns of monitored PA using data ascertained at age 60–64 years from a British birth cohort study.

## Methods

The Medical Research Council National Survey of Health and Development (NSHD) is a socially stratified sample of 5,362 singleton births that took place in 1 week of March 1946 in mainland Britain. Between 2006 and 2010 (at 60–64 years), 2,856 eligible study members (those known to be alive and living in England, Scotland, or Wales) were invited for assessment at one of six clinical research facilities (CRFs) or to be visited by a research nurse at home. Of these, 2,229 were assessed [1,690 at a CRF (15,16)]; invitations were not sent to those who had died (*n* = 778), who were living abroad (*n* = 570), had previously withdrawn from the study (*n* = 594) or had been lost to follow-up (*n* = 564).

Relevant ethical approval was provided in 2006–2010 by the Central Manchester Local Research Ethics Committee and the Scottish A Research Ethics Committee. All participants gave written informed consent.

### Assessment of Daily Patterns of PA at 60–64 Years

At the end of their clinical assessment, study participants were invited to wear a combined heart rate and acceleration monitor (Actiheart, CamNtech Ltd, Papworth, UK) attached in a standard position to their chest with two ECG-electrodes for five consecutive days (17). Participants were shown how to position the monitor by trained nurses and asked to wear it at all times, except when swimming and bathing. Heart rate and accelerometry counts were measured in 30-second epochs. Accelerometry counts were transformed by calculating log(1+count) at the half-minute level to normalize the distribution, reduce heteroscedasticity, and transform zero counts into zero measurements (18). The mean log-counts for each half-minute (ie, epoch) across days was calculated for each subject and a summary variable indicating the total daily log-activity counts was derived. Participants had to have a valid count measure recorded at each epoch across the 24-hour measurement period at least once during the 5 days of measurement; data were most likely to be missing during the night time. Missing data on counts at each epoch were imputed using the average of log-counts recorded for that participant at the same time epoch on other days. Any study participant with missing count data (with data assumed to be missing when both accelerometry count and heart rate for an epoch were 0) in the same time interval across all days was excluded; 9% of participants who had worn a monitor were excluded for this reason.

### BMI, Obesity History, and Body Composition

Heights and weights, self-reported at 26 years, and measured using a standard protocol by nurses at ages 36, 43, 53, and 60–64 were used to calculate BMI at each age [weight (kg)/height^2^ (m^2^)] and to categorize participants according to the age at first obesity (ie, BMI ≥30 kg/m^2^): never obese; or first obese by age: 26 or 36; 43; 53; 60–64. Current BMI was modeled as a continuous variable and also categorized into four standard groups: <25.0 kg/m^2^; 25.0–29.9 kg/m^2^; 30.0–34.9 kg/m^2^; ≥35.0 kg/m^2^. Too few participants (*n* = 7) were underweight (BMI < 18.5 kg/m^2^) to distinguish this group.

Measures of body composition were obtained for 1,658 of the 1,690 study participants who attended a CRF, in the supine position using a QDR 4500 Discovery DXA scanner (Hologic, Inc., Bedford, MA). Fat mass index was derived by dividing whole body fat mass (excluding the head) (kg) by height^2^ (m^2^).

### Covariates

Factors associated with BMI and PA were selected a priori as covariates: gender, occupational class, current work status, long-term limiting illness, symptoms of anxiety and depression, diabetes, and cardiovascular disease (17,19). Occupational class at age 53 (or if not available, the most recent measure in adulthood) was categorized using the Registrar General’s Social Classification of self-reported occupation into three groups: high (I or II), middle (IIINM or IIIM), and low (IV or V). Work status reported at age 60–64 was categorized into four groups: working full-time, working part-time, fully retired, and other. The presence of any long-term illness, health problem, or disability which limited activities was self-reported at age 60–64. The General Health Questionnaire-28 was used to identify those participants with symptoms of anxiety and depression at age 60–64 using a threshold of ≥5 to define cases (20). Self-reports of diabetes and doctor diagnosed angina and myocardial infarction from assessments undertaken up to and including age 60–64 were used to distinguish between participants with and without reports of diabetes and cardiovascular disease.

### Statistical Analyses

Linear regression models were used to test the associations of: (a) BMI at age 60–64 and (b) obesity history, with total daily log-activity counts. Initial models were adjusted for sex, with likelihood ratio tests of interactions of BMI and obesity history with sex undertaken. Subsequent models were adjusted for socioeconomic measures and markers of health status separately before both sets of covariates were adjusted together. When examining obesity history, BMI at age 60–64 was then included as a covariate in a final model. In initial models BMI at age 60–64 was modeled continuously, but where formal tests (involving inclusion of quadratic terms) indicated deviation from linearity analyses were rerun using BMI modeled categorically. To assess potential differences in the associations between BMI and PA in different segments of the day all models were then rerun to test associations of current BMI and obesity history with total log-activity counts in four segments of the day (morning [7 am–midday]; afternoon [midday–5 pm]; evening [5 pm–10 pm]; night [10 pm–7 am]). To formally assess differences by time of day, interactions of BMI, and obesity history with time of day (categorized into the four segments) were tested using likelihood ratio tests.

Analyses were performed using R version 3.2.2.

### Sensitivity Analyses

To confirm that any association found between BMI and PA was driven by adiposity, analyses were run to test the association of DXA-derived fat mass index with total daily log-activity counts in the subgroup of participants with this measure. To investigate whether associations with weight history, similar to those presented for age first obese, were also observed when using a lower threshold, the association of age at first overweight (ie, BMI ≥ 25.0 kg/m^2^) with total daily log-activity counts was examined.

## Results

Among the 1,639 participants with sufficient valid count measures to meet the inclusion criterion for analyses, the majority (*n* = 1,634) had data on current BMI. Of these, 1,388 had complete data on covariates and were included in the main analyses. The characteristics of participants are shown in Table 1.

**Table 1.** Characteristics of Participants From the MRC National Survey of Health and Development With Complete Data on Activity, BMI, and Covariates at 60–64 Years

		Mean (*SD*) or %	
		Men (*N* = 680)	Women (*N* = 708)
Activity counts			
Total daily log-activity count		2961.1 (844.5)	2943.7 (830.4)
Total daily activity count		43,088 (22,988)	41,619 (34,531)
Total log-activity count			
Morning (7am–midday)		976.2 (372.7)	947.4 (332.8)
Afternoon (midday–5 pm)		1022.9 (310.4)	1045.4 (306.5)
Evening (5 pm–10 pm)		636.1 (240.9)	658.9 (255.8)
Night (10 pm–7 am)		326.0 (230.5)	292.0 (226.7)
BMI and obesity history			
	BMI (kg/m^2^)	27.8 (4.1)	27.9 (5.3)
BMI (kg/m^2^)	<25.0*	26.6	33.1
	25.0–29.9	46.3	37.6
	30.0–34.9	21.5	19.4
	≥35.0	5.6	10.0
Age first obese (y)^†^	Never	68.4	64.9
	60–64	9.5	9.3
	53	11.6	13.2
	43	5.5	6.5
	26 or 36	5.0	6.1
Covariates			
Occupational class	High (I or II)	59.3	41.4
	Middle (IIINM or IIIM)	31.8	43.6
	Low (IV or V)	9.0	15.0
Work status	Working full-time	50.3	14.7
	Working part-time	16.3	28.4
	Fully retired	32.4	55.9
	Other^‡^	1.0	1.0
Long-term limiting illness		21.6	22.0
Symptoms of anxiety and depression (GHQ-28 score ≥ 5)		11.1	20.6
Cardiovascular disease		8.8	3.4
Diabetes		7.2	6.1

*Note:* BMI = body mass index; *SD* = standard deviation. *BMI < 18.5 kg/m^2^: 2 men, 5 women. ^†^ *N* = 1,115 due to missing data on BMI at earlier ages. ^‡^Includes those who classify themselves as housewives or unemployed (ie, not currently working but seeking employment).

Current BMI was strongly associated with total daily activity at age 60–64. When BMI was modeled continuously (Supplementary Table S1) there was evidence of deviation from linearity (*p* = .04), thus BMI was modeled categorically in the main analyses (Table 2). In preliminary sex-adjusted models, those participants with BMI ≥30 kg/m^2^ had lower mean total daily log-activity counts than those with BMI <25 kg/m^2^, with clear evidence of a graded association across the two categories of BMI above 30 kg/m^2^. This association was only partially attenuated after adjustment for health status and socioeconomic measures; in a fully-adjusted model, participants with BMI ≥35 kg/m^2^ had a mean total daily (log) activity count 581.7 units lower (95% confidence interval [CI]: −757.2, −406.3) than those with BMI <25 kg/m^2^, representing an 18.4% difference.

**Table 2.** Associations of Current BMI and Obesity History With Total Daily Log-Activity Counts at Age 60–64

	**Mean Difference in Total Daily Log-Activity Counts (95% CI)**				
**Model Adjusted for:**	**1: Sex**	**2: Sex and Socioeconomic Factors**	**3: Sex and Health Status**	**4: All Covariates in Models 2 and 3**	5: All Covariates in Model 4 Plus Current BMI*
Current BMI (kg/m^2^) [*N* = 1,388]					
<25.0^†^	0	0	0	0	—
25.0–29.9	−91.4 (−194.9, 12.2)	−91.4 (−194.8, 12.0)	−77.8 (−180.4, 24.9)	−79.4 (−182.0, 23.2)	
30.0–34.9	−282.9 (−406.9, −159.0)	−284.6 (−408.6, −160.7)	−228.8 (−353.2, −104.4)	−232.0 (−356.6, −107.4)	
≥35.0	−671.6 (−844.5, −498.6)	−669.3 (−843.4, −495.2)	−570.1 (−744.7, −395.4)	−581.7 (−757.2, −406.3)	
*p*-Value for overall association	<.01	<.01	<.01	<.01	
Age first obese (y) [*N* = 1,115]					
Never	0	0	0	0	0
60–64	−200.6 (−371.1, −30.0)	−204.1 (−375.2, −33.0)	−149.9 (−320.2, 20.4)	−155.4 (−326.6, 15.7)	45.2 (−150.9, 241.3)
53	−261.1 (−412.8, −109.4)	−255.9 (−407.6, −104.1)	−225.5 (−377.3, −73.6)	−225.4 (−377.3, −73.5)	10.2 (−179.4, 199.8)
43	−449.9 (−658.7, −241.2)	−439.7 (−648.6, −230.8)	−364.1 (−576.5, −151.8)	−360.6 (−573.1, −148.2)	−62.0 (−318.3, 194.4)
26 or 36	−524.4 (−740.8, −308.0)	−518.4 (−739.5, −297.3)	−398.8 (−620.3, −177.3)	−413.1 (−638.1, −188.2)	−27.2 (−319.3, 264.9)
*p*-Value for trend	<.01	<.01	<.01	<.01	.71

*Notes:* BMI = body mass index; CI = confidence interval. Model 1: adjusted for sex (tests of sex interaction, *p* = .23 [current BMI]; 0.41 [obesity history]). Model 2: sex, occupational class, and work status. Model 3: sex, long-term limiting illness, symptoms of anxiety and depression, cardiovascular disease, and diabetes. Model 4: adjusted for all covariates in models 2 and 3. *Effect estimate per 1 kg/m^2^ increase in BMI adjusted for obesity history and other covariates (*N* = 1,115): −34.0 (−50.6, −17.4). ^†^See Supplementary Table S5 for results from sensitivity analyses excluding those seven participants with BMI <18.5 kg/m^2^.

The younger the age when first obese, the lower the mean total daily activity (*p* < .01, test for trend; Table 2). These associations were only partially attenuated after adjustment for health status and socioeconomic measures; mean difference in total daily (log) activity counts = −413.1 (CI: −638.1, −188.2) when comparing those who were first obese at 26 or 36 years with those who were never obese, representing a 13.1% difference. Associations were fully attenuated after adjustment for current BMI.

**Figure 1.**
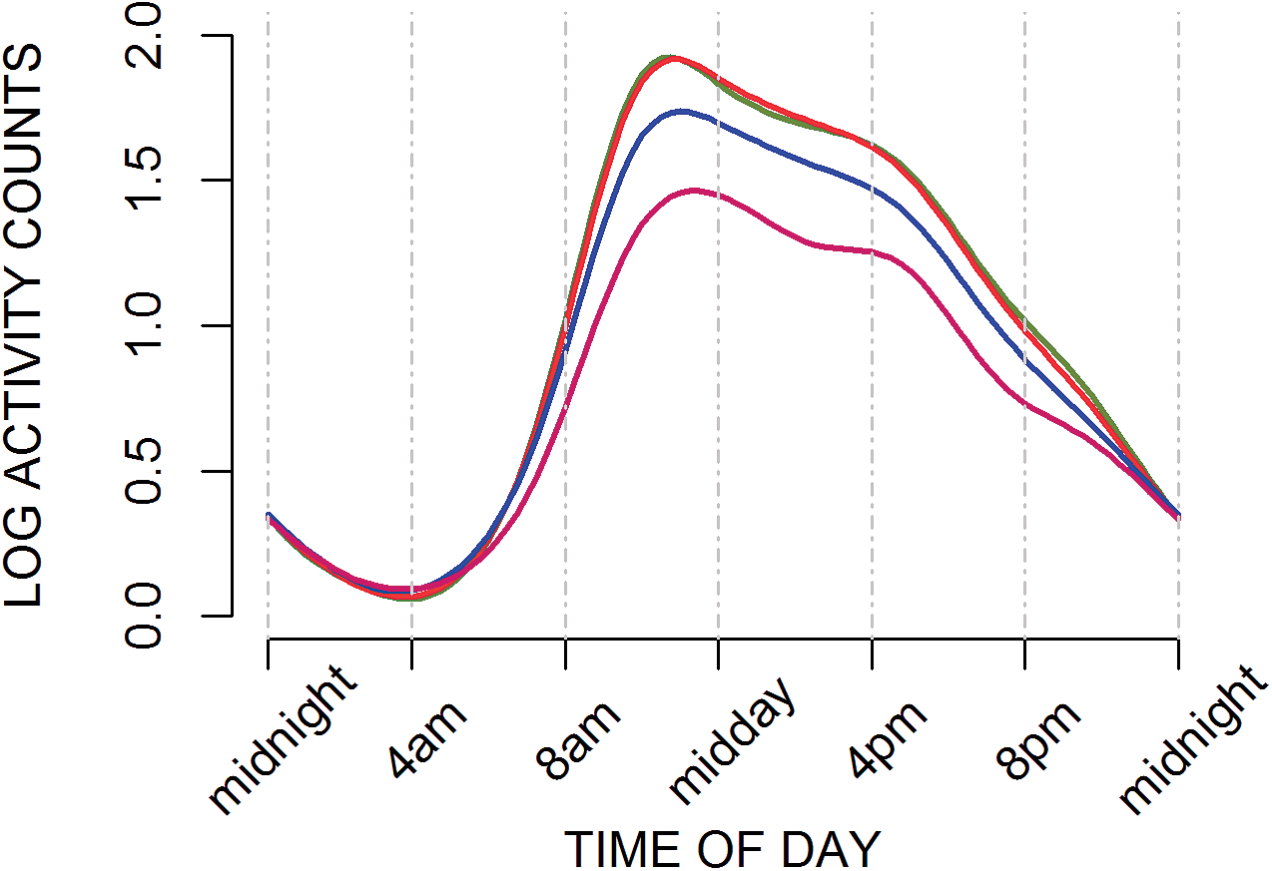
Smoothed curves of mean log-activity counts at age 60–64 by current BMI (*N* = 1,388). BMI (kg/m^2^) categories: <25.0 (green); 25.0–29.9 (red); 30.0–34.9 (blue); ≥35.0 (pink).

**Figure 2.**
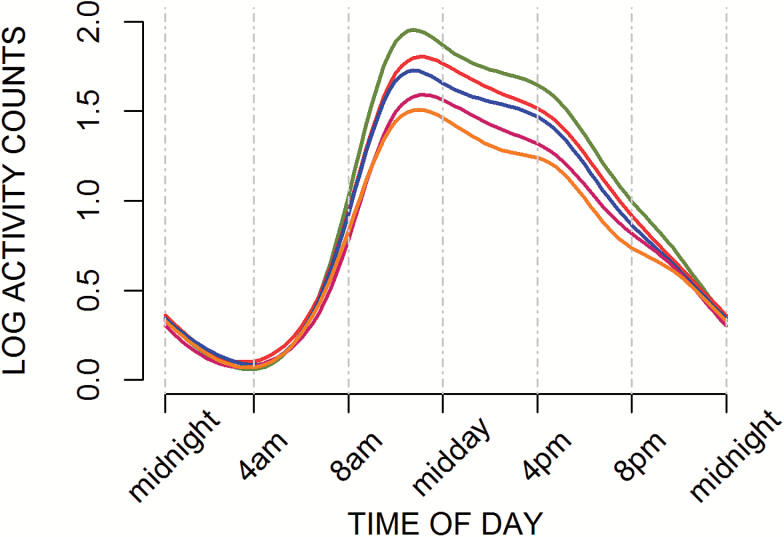
Smoothed curves of mean log-activity counts at age 60–64 by obesity history (*N* = 1,115). Categories: never obese (green); first obese at: 60–64 y (red); 53 y (blue); 43 y (pink); 26 or 36 y (yellow).

Daily patterns of PA were visually explored (Figures 1 and 2) and formally assessed using time of the day-specific tests (Supplementary Tables S2 and S3). Higher current BMI and younger age at obesity were associated with lower levels of activity in all segments of the day with the exception of overnight (ie, 10 pm–7 am). Despite some variation in the size of effect estimates in different segments of the day (Supplementary Tables S2 and S3), there were no statistically significant interactions between BMI or obesity history and time of day when the overnight segment was excluded.

Sensitivity analyses found slightly stronger associations with fat mass index than BMI. Similar, but slightly weaker, associations for age at first overweight were found when compared with results for age at first obese (Supplementary Table S1).

## Discussion

In a British birth cohort study, current BMI and obesity history were strongly associated with daily patterns of activity at 60–64 years. Participants classified as obese (BMI ≥ 30 kg/m^2^) had lower levels of activity across all segments of the day when compared with participants with a healthy BMI (ie, <25 kg/m^2^) except overnight. Within the group who were classified as obese at age 60–64, higher BMI and younger age at onset of obesity (reflecting greater length of exposure) were both associated with lower levels of daily activity. These associations were only partially attenuated after adjustment for a range of covariates, including long-term limiting illness and chronic conditions; for example, there remained a 13.1% difference in daily activity levels when comparing those never obese with those obese from age 26 or 36 after adjustments.

The novel contribution of our study is in investigating the role of obesity history in relation to daily patterns of monitored activity. In the AGESII-Reykjavik study, obesity in midlife was found to be associated with sedentariness, assessed by acceleromery, in old age [age 73–92 years (21)]. However, our findings using repeated measures of BMI across adulthood are the first to show a cumulative detrimental effect of obesity on PA levels later in life.

The finding of a strong cross-sectional association between BMI and daily activity in the NSHD is consistent with findings from other studies of older populations which have investigated the role of a range of factors in explaining diurnal variation in activity levels. These found BMI to be one of the key factors that remained significant in multivariate models (22,23). As in our study, findings from the British Regional Heart Study (BRHS), comprising men aged 71–91 years, seem to suggest that this association may be nonlinear, with more marked differences in daily patterns of activity observed with increasing obesity (22).

That the association of obesity history with activity was attenuated after adjustment for current BMI does not negate the importance of obesity history as this can be explained by the fact that, as BMI tracks across life, BMI at age 60–64 acts as a marker of weight history. Those people who were first recorded as obese in earlier adulthood are likely to have higher mean BMI at age 60–64 than those recorded as obese for the first time at this age. This is demonstrated by observing that mean BMI at age 60–64 among those who were obese by 26/36 was 36.8 (standard deviation [*SD*] = 5.8) compared with 31.4 (*SD* = 1.3) among those first obese at 60–64.

Greater length of exposure to obesity across adulthood is associated with increased risk of a range of health and functional outcomes in later life, including low muscle quality, osteoarthritis, and mobility limitations (24–26). Recent work has shown that mobility limitations and chronic diseases are associated with less favorable diurnal patterns of activity in older men (22). Similarly, the current findings also demonstrate that markers of health status were most likely to attenuate the effect of BMI on PA with long-term limiting illness and diabetes having the greatest impact. In addition to greater disease burden, psychological and other barriers to PA faced by those who are obese also need to be considered (27,28).

Declining PA levels with increasing age are at least partially driven by declines in energy availability. This is linked to the changing energetic demands of movement which have been shown to increase with age and also BMI (29,30). At any given age, people classified as obese will, on average, require more energy to undertake a given weight-bearing activity (such as walking) than those people with lower BMI (31). It is possible that people who are obese, especially those who have been obese for longer, are therefore less active throughout the day because they are more likely to become fatigued. In the Baltimore Longitudinal Study of Aging, it was suggested that the marked drop in activity levels observed in participants aged 68+ years from the early afternoon onwards [which has also been observed in the BRHS (22)] may be explained by fatigue (23). While the same phenomenon is not seen among obese participants in the NSHD, this may be attributed to the younger age of participants whose daily routines are still more likely to be influenced by fixed patterns of employment. The role of fatigue warrants further investigation as the cohort ages.

Key strengths of our analyses include the use of a relatively large sample of men and women of the same age on whom prospective data on BMI and covariates have been collected across life. In addition, our measure of PA captures all movement including light intensity and nonvolitional activities common in older age which are typically unreliable when assessed via self-report (32). This method of ascertainment also avoids the bias that may be introduced by differential mis-reporting of activity by weight status.

By focusing on total daily counts, a proxy of total volume of PA (5), we have been able to study activity across the full range of intensity, whereas application of intensity cut-points makes assumptions which may introduce bias and limit comparability of findings across studies. This approach also ensures our findings are comparable with those from other studies which have used different accelerometers (eg, Actigraph). Despite the many benefits of the data used it is necessary to consider that activity was monitored over 5 days and so may not be representative of usual activity over the course of a year. In addition, these data do not provide information on the type and context of activities being undertaken.

In deriving a measure of obesity history it was assumed that once a person was obese they remained so. While this has limitations because weight loss can occur and may be associated with different patterns of activity, we did find that our assumption was reasonable; of those first obese between ages 26 and 53, 78% were obese at all subsequent ages of assessment. Further, relationships were largely similar when those not consistently obese were examined separately (Supplementary Table S4). The study population was selected at birth to be nationally representative and despite losses to follow-up has remained so, in many respects, at age 60–64 (16). It is acknowledged that some bias may have been introduced due to these losses, especially as some will be from death caused by weight-related illnesses. Another potential source of bias is the restriction of analyses to those with complete data. However, bias introduced by these sample restrictions is expected to be minimal; there were no differences in activity levels or BMI when those participants with complete data were compared to those with missing data.

Our findings that current BMI and obesity history were strongly associated with daily patterns of activity at 60–64 years have important public health implications. As a consequence of the obesity epidemic, younger generations have accrued more time exposed to obesity across their lifetimes, and so the proportion of older adults achieving sufficient levels of activity to realize its health benefits is likely to decline. Opportunities to break this cycle need to be taken as early in life as possible as the associations between obesity and inactivity are likely to become stronger the longer they have been established. Our findings also suggest that older adults who are obese may require additional support and targeted interventions to increase their daily levels of PA.

## Supplementary Material

Supplementary data are available at *The Journals of Gerontology, Series A: Biological Sciences and Medical Sciences* online.

## Funding

R.C., R.H., and D.K. are supported by the UK Medical Research Council (Programme codes: MC_UU_12019/1, MC_UU_12019/2, and MC_UU_12019/4). L.H. and C.C. are supported by grants from the National Heart, Lung and Blood Institute (5R01HL123407-02) and the National Institute of Neurological Disorders and Stroke (5R01NS060910-07). J.A.S. is supported by the National Institute on Aging (K01AG048765 and HHSN311210300177P). T.H. is supported by the National Institute on Aging, Intramural Research Program. The MRC National Survey of Health and Development is funded by the UK Medical Research Council.

## Conflict of Interest

None declared.

## Supplementary Material

Supplementary Material
