# Supplementary material for: Obesity History and Daily Patterns of Physical Activity at Age 60–64 Years: Findings From the MRC National Survey of Health and Development
Source: J Gerontol A Biol Sci Med Sci. 2017 Feb 18;72(10):1424–30. doi: 10.1093/gerona/glw331 (PMC5861926; doi:10.1093/gerona/glw331)
Supplement: Supplementary Material [file glw331_suppl_supplementary_material.docx]

**Table S1: Associations of current BMI, fat mass index and history of overweight with total daily log activity counts at age 60-64 years in the MRC National Survey of Health and Development**

|  | **Mean difference in total daily log activity counts (95% CI)** | | | |
| --- | --- | --- | --- | --- |
| **Model adjusted for:** | **1: sex** | **2: sex & socioeconomic factors** | **3: sex & health status** | **4: all covariates** |
| Body mass index, per 1kg/m^2^ (N=1388) | -39.6 (-48.6, -30.5) | -39.6 (-48.7, -30.5) | -33.1 (-42.4, -23.8) | -33.8 (-43.1, -24.5) |
|  |  |  |  |  |
| Fat mass index, per 1kg/m^2^ (N=1081) | -77.6 (-93.1, -61.9) | -77.5 (-93.1, -61.9) | -69.7 (-85.5, -53.8) | -70.2 (-86.0, -54.4) |
|  |  |  |  |  |
| Age first overweight (y) (N=1148)  *Never*  *60-64*  *53*  *43*  *36*  *26* | 0  -74.1 (-274.2, 126.0)  -63.2 (-213.8, 87.5)  -138.3 (-305.0, 28.4)  -313.8 (-472.0, -155.6)  -284.5 (-438.4, -130.5) | 0  -77.7 (-277.6, 122.1)  -68.0 (-218.5, 82.6)  -132.8 (-299.4, 33.8)  -315.2 (-473.4, -157.1)  -281.2 (-436.1, -126.3) | 0  -71.0 (-268.1, 126.0)  -34.6 (-183.3, 114.0)  -116.9 (-81.6, 47.7)  -262.5 (-419.2, -105.8)  -183.2 (-339.2, -27.3) | 0  -72.6 (-269.8, 124.6)  -39.8 (-188.6, 109.1)  -110.7 (-275.6, 54.2)  -269.5 (-426.4, -112.6)  -191.0 (-347.8, -34.1) |

Model 1: adjusted for sex [BMI: formal test of deviation from linearity, p=0.04; fat mass index: formal test of deviation from linearity, p<0.01]

Model 2: model 1 plus occupational class and work status

Model 3: model 1 plus long-term limiting illness, symptoms of anxiety and depression, cardiovascular disease and diabetes

Model 4: adjusted for all covariates in models 2 and 3

As for age first obese, associations with age first overweight attenuated after adjustment for current BMI

**Table S2: Associations of current BMI with total log activity counts at age 60-64 years in different segments of the day in the MRC National Survey of Health and Development (N=1388)**

|  |  | **Mean difference in total log activity counts in each segment (95% CI)** | |
| --- | --- | --- | --- |
| **Segment of the day** | **BMI (kg/m^2^)** | **Model 1: sex-adjusted** | **Model 2: fully-adjusted** |
| Morning (7am – midday) | *< 25.0*  *25.0-29.9*  *30.0-34.9*  *≥ 35.0*  p-value | 0  -43.3 (-87.1, 0.5)  -119.0 (-171.4, -66.6)  -261.2 (-334.4, -188.1)  <0.01 | 0  -40.0 (-83.4, 3.3)  -102.6 (-155.3, -50.0)  -229.6 (-303.6, -155.5)  <0.01 |
|  |  |  |  |
| Afternoon (midday – 5pm) | *< 25.0*  *25.0-29.9*  *30.0-34.9*  *≥ 35.0*  p-value | 0  -23.6 (-61.8, 14.7)  -101.3 (-147.1, -55.6)  -231.4 (-295.2, -167.6)  <0.01 | 0  -18.9 (-56.7, 18.9)  -78.6 (-124.6, -32.7)  -197.8 (-262.5, -133.2)  <0.01 |
|  |  |  |  |
| Evening (5pm – 10pm) | *< 25.0*  *25.0-29.9*  *30.0-34.9*  *≥ 35.0*  p-value | 0  -34.5 (-65.4, -3.6)  -87.1 (-124.1, -50.1)  -178.3 (-229.9, -126.7)  <0.01 | 0  -31.4 (-62.1, -0.6)  -77.0 (-114.4, -39.7)  -159.3 (-211.9, -106.7)  <0.01 |
|  |  |  |  |
| Night (10pm – 7am) | *< 25.0*  *25.0-29.9*  *30.0-34.9*  *≥ 35.0*  p-value | 0  10.0 (-18.9, 39.0)  24.5 (-10.2, 59.1)  -0.6 (-49.0, 47.7)  0.55 | 0  10.9 (-18.1, 39.9)  26.4 (-8.8, 61.6)  5.0 (-44.6, 54.6)  0.53 |

Model 1: adjusted for sex

Model 2: adjusted for sex, occupational class, work status, long-term limiting illness, symptoms of anxiety and depression, cardiovascular disease and diabetes

p-values presented are for overall tests of association

**Table S3: Associations of obesity history with total log activity counts at age 60-64 years in different segments of the day in the MRC National Survey of Health and Development (N=1115)**

|  |  | **Mean difference in total log activity counts in each segment (95% CI)** | |
| --- | --- | --- | --- |
| **Segment of the day** | **Age first obese (y)** | **Model 1: sex-adjusted** | **Model 2: fully-adjusted** |
| Morning (7am – midday) | *Never*  *60-64*  *53*  *43*  *26 or 36*  p-value | 0  -93.7 (-165.3, -22.1)  -111.9 (-175.6, -48.2)  -159.8 (-247.5, -72.2)  -153.3 (-243.5, -63.2)  <0.01 | 0  -77.6 (-149.2, -6.0)  -100.2 (-163.7, -36.6)  -130.4 (-219.3, -41.5)  -144.2 (-207.9, -20.5)  <0.01 |
|  |  |  |  |
| Afternoon (midday – 5pm) | *Never*  *60-64*  *53*  *43*  *26 or 36*  p-value | 0  -81.8 (-144.4, -19.1)  -110.1 (-165.9, -54.4)  -160.7 (-237.4, -84.1)  -198.1 (-277.0, -119.2)  <0.01 | 0  -57.0 (-119.7, 5.7)  -98.2 (-153.8, -42.6)  -128.2 (-206.0, -50.4)  -159.1 (-241.1, -77.2)  <0.01 |
|  |  |  |  |
| Evening (5pm – 10pm) | *Never*  *60-64*  *53*  *43*  *26 or 36*  p-value | 0  -61.7 (-112.1, -11.3)  -79.7 (-124.5, -34.8)  -113.9 (-175.6, -52.2)  -158.2 (-221.7, -94.8)  <0.01 | 0  -53.4 (-104.3, -2.6)  -72.0 (-117.1, -26.8)  -99.5 (-162.6, -36.4)  -134.3 (-200.8, -67.8)  <0.01 |
|  |  |  |  |
| Night (10pm – 7am) | *Never*  *60-64*  *53*  *43*  *26 or 36*  p-value | 0  36.6 (-12.2, 85.4)  40.6 (-2.8, 84.0)  -15.5 (-75.2, 44.3)  -17.5 (-79.0, 44.0)  0.83 | 0  32.6 (-16.9, 82.0)  44.9 (1.0, 88.8)  -2.6 (-65.0, 58.8)  -4.8 (-69.5, 59.9)  0.45 |

Model 1: adjusted for sex

Model 2: adjusted for sex, occupational class, work status, long-term limiting illness, symptoms of anxiety and depression, cardiovascular disease and diabetes

p-values presented are for tests of trend

**Table S4: Associations of obesity history with total daily log-activity counts at age 60-64 years in the MRC National Survey of Health and Development (N=1115) [those not consistently obese distinguished from those obese at all subsequent ages of assessment]**

|  | **Mean difference in total daily log-activity counts (95% CI)** | | | |  |
| --- | --- | --- | --- | --- | --- |
| **Model adjusted for:** | **1: sex** | **2: sex & socioeconomic factors** | **3: sex & health status** | **4: all covariates in models 2 and 3** | **5: all covariates in model 4 plus current BMI** |
| Age first obese (y)  *Never (n=743)*  *Not consistently obese^*^ (n=59)*  *60-64 (n=105)*  *53 (n=110)*  *43 (n=50)*  *26 or 36 (n=48)*  *p-value for overall association* | 0  -116.7 (-337.5, 104.0)  -200.6 (-370.8, -30.4)  -331.3 (-498.1, -164.6)  -592.0 (-830.6, -353.5)  -538.1 (-781.6, -294.6)  <0.01 | 0  -84.8 (-306.7, 137.1)  -204.4 (-375.0, -33.8)  -329.6 (-496.1, -163.1)  -590.3 (-828.6, -352.0)  -540.0 (-789.8, -290.1)  <0.01 | 0  -34.3 (-258.3, 189.7)  -149.4 (-319.2, 20.5)  -301.8 (-467.6, -135.9)  -501.5 (-741.5, -261.4)  -391.4 (-640.1, -142.6)  <0.01 | 0  -16.4 (-241.3, 208.5)  -155.9 (-326.4, 14.7)  -302.5 (-468.2, -136.7)  -506.1 (-746.0, -266.1)  -416.3 (-670.1, -162.5)  <0.01 | 0  109.8 (-126.5, 346.0)  19.6 (-180.0, 219.1)  -70.2 (-285.8, 145.5)  -191.9 (-495.8, 112.0)  -47.8 (-383.0, 287.3)  0.58 |

Model 1: adjusted for sex

Model 2: sex, occupational class and work status

Model 3: sex, long-term limiting illness, symptoms of anxiety and depression, cardiovascular disease and diabetes

Model 4: adjusted for all covariates in models 2 and 3

* Classified as first obese at age 26, 36, 43 or 53 but not classified as obese at all subsequent ages of assessment

**Table S5: Association of current BMI with total daily log-activity counts at age 60-64 in the MRC National Survey of Health and Development after exclusion of those 7 participants with BMI<18.5kg/m^2^ (N=1381)**

|  | **Mean difference in total daily log-activity counts (95% CI)** | | | |
| --- | --- | --- | --- | --- |
| **Model adjusted for:** | **1: sex** | **2: sex & socioeconomic factors** | **3: sex & health status** | **4: all covariates in models 2 and 3** |
| Current BMI (kg/m^2^)  *18.5-25.0*  *25.0-29.9*  *30.0-34.9*  *≥35.0*  *p-value for overall association* | 0  -81.7 (-185.7, 22.2)  -273.3 (-397.5, -149.1)  -661.8 (-834.8, -488.8)  <0.01 | 0  -83.4 (-187.2, 20.4)  -277.2 (-401.4, -153.0)  -664.4 (-838.8, -490.1)  <0.01 | 0  -68.2 (-171.3, 34.8)  -219.8 (-344.4, -95.1)  -561.7 (-736.5, -386.9)  <0.01 | 0  -71.5 (-174.5, 31.5)  -225.1 (-349.9, -100.3)  -578.0 (-753.7, -402.3)  <0.01 |

Model 1: adjusted for sex

Model 2: sex, occupational class and work status

Model 3: sex, long-term limiting illness, symptoms of anxiety and depression, cardiovascular disease and diabetes

Model 4: adjusted for all covariates in models 2 and 3
